# Body Mass Index Within Multifactor Predictors of Ventral Hernia Recurrence: A Retrospective Cohort Study

**DOI:** 10.7759/cureus.41148

**Published:** 2023-06-29

**Authors:** Abdulwahab H Alansari, Asim M Almalawi, Abdullah Alghamdi, Mohammed S Alghamdi, Hassan A Hazazi, Ahmed A Aljabri, Raed A Alsulami, Abdulaziz M Alkhoshi, Fatma Khinaifis

**Affiliations:** 1 General Surgery, King Abdulaziz University Hospital, Jeddah, SAU; 2 Infectious Diseases, King Abdulaziz University Hospital, Jeddah, SAU; 3 Family, King Abdulaziz University Hospital, Jeddah, SAU; 4 Pathology, King Abdulaziz University Hospital, Jeddah, SAU; 5 Internal Medicine, King Abdulaziz University Hospital, Jeddah, SAU; 6 Neurosurgery, King Abdulaziz University Hospital, Jeddah, SAU; 7 Surgical Oncology, King Abdulaziz University Hospital, Jeddah, SAU

**Keywords:** surgical repair, type of hernia, obesity, body-mass index, hernia recurrence

## Abstract

Background

A ventral hernia is a protrusion of the peritoneum through the defective abdominal wall. Several risk factors increase the likelihood of hernial recurrence. One of the most common risk factors is obesity, defined by the World Health Organization (WHO) as increased body mass index (BMI). Few studies have explored the effects of BMI and other factors on hernia recurrence. Hence, we aimed to investigate the role of increased BMI in hernia recurrence in conjunction with various risk factors such as age, sex, type of hernia, the time elapsed between the occurrence and recurrence, complications of hernia, and procedure.

Methods

This retrospective cohort study was conducted at King Abdulaziz University Hospital (KAUH). All the patients were admitted between 2015-2022. A total of 1676 medical records were obtained from all patients who underwent hernia repair more than once or were diagnosed with a recurrent hernia during the study period.

Results

Our study revealed an insignificant correlation between a BMI of more than 25 kg/m^2^ and the recurrence of inguinal hernias, predominantly indirect hernias. Furthermore, overweight and obese patients experience a longer interval between the first and second hernia repairs. Interestingly, all the patients with inguinal and umbilical hernias had the same diagnosis at the second presentation. However, the findings also included a significant increase in umbilical hernias in individuals with a high BMI and higher recurrence rates among male patients with inguinal hernias.

Conclusion

BMI higher than 25 kg/m^2^ increases recurrence rates for umbilical hernias but decreases the recurrence of inguinal hernias.

## Introduction

Worldwide, patients who undergo ventral hernia repair have a recurrence rate of up to 40% [1]. A ventral hernia is a protrusion of the omentum or peritoneal content through a defect in the abdominal wall. It includes various types, the most frequently seen of which are inguinal, incisional, and umbilical [1]. Moreover, It has been demonstrated that several risk factors increase the likelihood of hernia recurrence: First, hernia-related factors like the hernia's diameter or location; Second, factors related to the surgery itself such as the type of anesthesia used, suture used, and type of repair performed; Third, the likelihood of postoperative infection, hematoma, and patient-related factors such as sex, weight, genetics, lifestyle factors, medications, elevated intra-abdominal pressure, immunosuppression, and other comorbidities [2-4]. Additionally, One of the most important risk factors is obesity, which is defined according to the World Health Organization (WHO) by body mass index (BMI), an anthropometric measurement used to categorize individuals into subclasses [5]. Obesity is considered a risk factor for an increased recurrence rate of hernias [6]. However, it is a growing health issue in modern societies, people with severe obesity, especially those with a BMI of 35.0 to 39.9 (class II) or greater than 40 (class III), are more likely to experience health problems and die prematurely. Obesity is a known risk factor for a number of chronic diseases, including cardiovascular disease, type 2 diabetes, cancer, asthma, osteoarthritis, chronic back pain, obstructive sleep apnea, non-alcoholic fatty liver disease, and gallbladder diseases [7,8]. Several studies have suggested that obesity is a significant comorbidity for hernia recurrence [9-12].

In contrast, an American study reported the opposite results [10]. However, to the best of our knowledge, few studies have explored the effect of BMI on several other factors such as age, sex, type of hernia, surgical repair, and the period between the first repair and the second diagnosis of hernia recurrence. Even though recurrent hernias subject patients to multiple operations regardless of the hernia repair procedure, patients are exposed to a higher risk of surgical complications [13]. Hence, we aimed to investigate the role of BMI in hernia recurrence, in conjunction with various risk factors.

## Materials and methods

Study design

This retrospective cohort study was conducted at King Abdulaziz University Hospital (KAUH) in Jeddah, Saudi Arabia. Involves all the patients who have been admitted 2015-2022 to undergo hernia repair. This study was approved by the KAUH Ethical Committee (reference number 527-22). Data for the study were obtained from the Hospital Health Informatics Department, collected using a data collection sheet, and interpreted using a Microsoft Excel spreadsheet.

The medical records of 1676 patients were reviewed. All patients who underwent hernia repair more than once or were diagnosed with a recurrent hernia at KAUH during the study period were included. Patients aged less than two years were excluded from the study.

Patient demographics included age at the time of surgery, sex, and BMI at the time of the first and second operation or diagnosis. Data regarding the types of hernia, such as inguinal, umbilical, femoral, incisional, or obstructed, repair with mesh or non-mesh, date of operations, or diagnoses, were collected.

Patients were classified according to age into pediatrics if they were aged <16 years, adults aged 16-50 years old, and elderly aged > 50 years. Additionally, BMI was defined as ≥ 25 kg/m^2^ as overweight or obese and < 25 as normal weight.

Statistical analysis was carried out using RStudio (R version 4.1.1; R Core Team (2022). R: A language and environment for statistical computing. R Foundation for Statistical Computing, Vienna, Austria). Categorical variables are expressed using frequencies and percentages, whereas numerical variables are presented as the median and interquartile range (IQR). Statistical differences between different groups were assessed using Pearson’s chi-squared test or Fisher’s exact test for categorical data or a Wilcoxon rank-sum test for continuous data. Changes in the type of hernia over time were assessed using McNemar’s chi-squared test with continuity correction.

## Results

Demographic and hernia-related characteristics

In the current study, we analyzed the data of 98 patients who had recurrent hernias. More than two-thirds of patients (67.3%) were males. More than half of the patients were aged > 50 years (51.0%), whereas 37.8% and 11.2% of them were aged 16 to 50 and < 16 years, respectively. At the time of the first intervention, the inguinal hernia was the most common type of hernia (72.4%) while 18.4% and 9.2% of patients had umbilical and incisional hernias, respectively. The obstructed hernia was prevalent among 25.5%, and mesh repair was performed among 68.4% of patients (Table 1).

**Table 1:** Factors associated with the incidence of a recurrent hernia

Parameter	Category	Umbilical Hernia			Inguinal Hernia			Incisional Hernia		
		No, N = 79	Yes, N = 19	p-value	No, N = 30	Yes, N = 68	p-value	No, N = 87	Yes, N = 11	p-value
Gender	Male	64 (81.0%)	2 (10.5%)	<0.001	5 (16.7%)	61 (89.7%)	<0.001	63 (72.4%)	3 (27.3%)	0.005
	Female	15 (19.0%)	17 (89.5%)		25 (83.3%)	7 (10.3%)		24 (27.6%)	8 (72.7%)	
Age at second surgery	< 16 y	9 (11.4%)	1 (5.3%)	0.246	1 (3.3%)	9 (13.2%)	0.210	10 (11.5%)	0 (0.0%)	0.644
	16 to 50 y	21 (26.6%)	9 (47.4%)		12 (40.0%)	18 (26.5%)		27 (31.0%)	3 (27.3%)	
	> 50 y	49 (62.0%)	9 (47.4%)		17 (56.7%)	41 (60.3%)		50 (57.5%)	8 (72.7%)	
Obstructed	Yes	24 (30.4%)	1 (5.3%)	0.037	3 (10.0%)	22 (32.4%)	0.019	23 (26.4%)	2 (18.2%)	0.724
Type of surgical repair at the first operation	Mesh	56 (70.9%)	11 (57.9%)	0.415	16 (53.3%)	51 (75.0%)	0.030	62 (71.3%)	5 (45.5%)	0.045
	Non-mesh herniorrhaphy	22 (27.8%)	8 (42.1%)		13 (43.3%)	17 (25.0%)		25 (28.7%)	5 (45.5%)	
	Not specified	1 (1.3%)	0 (0.0%)		1 (3.3%)	0 (0.0%)		0 (0.0%)	1 (9.1%)	
BMI at second surgery	BMI < 25	30 (38.0%)	2 (10.5%)	0.022	2 (6.7%)	30 (44.1%)	<0.001	32 (36.8%)	0 (0.0%)	0.014
	BMI 25 or more	49 (62.0%)	17 (89.5%)		28 (93.3%)	38 (55.9%)		55 (63.2%)	11 (100.0%)	
Time between surgeries	Months	24.5 (14.0, 60.0)	17.6 (11.1, 54.5)	0.486	22.2 (14.9, 54.5)	23.9 (12.4, 59.8)	0.982	22.9 (12.4, 59.1)	35.0 (17.2, 55.5)	0.362

Baseline differences based on BMI groups

At the time of the first presentation, a total of 69 patients (70.4%) were overweight or obese (had a BMI of ≥25 kg/m^2^). A significantly higher proportion of patients with high BMI levels (≥25 kg/m^2^) aged > 50 years (55.1% vs 41.4% had BMI <25 kg/m^2^, p < 0.001). Additionally, patients with high BMI levels (≥25 kg/m^2^) had a significantly higher rate of umbilical hernia (24.6% vs 3.4%, p = 0.013), as well as lower rates of inguinal hernia (62.3% vs 96.6%, p < 0.001) and indirect hernia (31.9% vs 69.0%, p < 0.001, Table 1).

Changes in the types of hernias over time 

**Figure 1:**
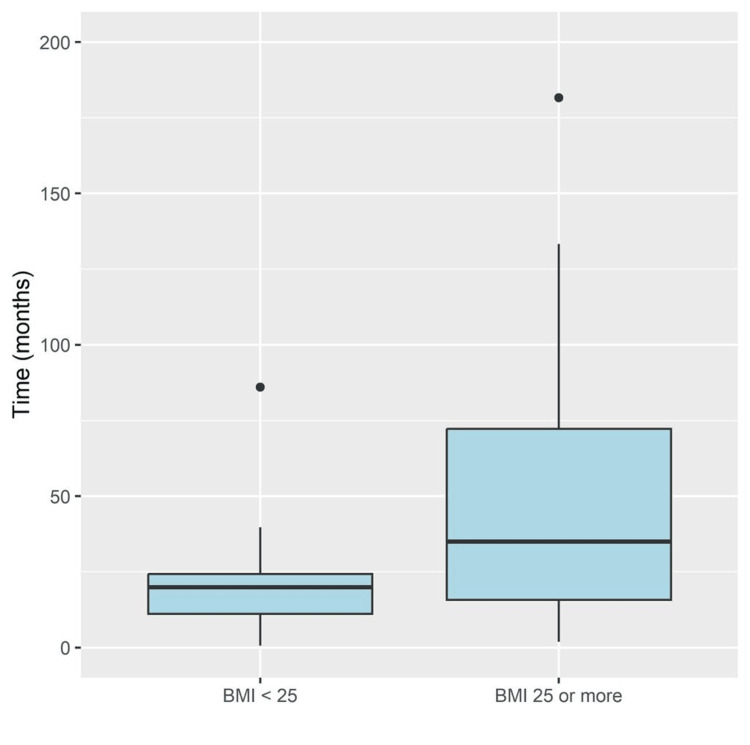
Changes in the types of hernias over time

The median time between the first and second interventions for hernia repair was 23.2 months (13.6, 59.3). Overweight and obese patients had a significantly longer time between the first and second interventions (median = 35.0 months, IQR = 15.8 to 72.2) compared to other patients (median = 19.8 months, IQR = 11.2 to 24.3, p = 0.006, Figure 1). The majority of patients who had umbilical and inguinal hernias at the first intervention had the same diagnosis at the second intervention (94.4% for both types of hernias), whereas all the patients who had baseline incisional hernias had the same diagnosis at the second presentation. There were no significant differences in the type of hernias over time (from the first to second interventions, Table 2).

**Table 2:** Temporal changes in the types of hernias

Hernia at the first intervention	Hernia at the second intervention		p
Umbilical hernia	No, N = 79	Yes, N = 19	
No	78 (97.5%)	2 (2.5%)	>0.999
Yes	1 (5.6%)	17 (94.4%)	
Inguinal hernia	No, N = 30	Yes, N = 68	
No	26 (96.3%)	1 (3.7%)	0.371
Yes	4 (5.6%)	67 (94.4%)	
Incisional hernia	No, N = 87	Yes, N = 11	
No	87 (97.8%)	2 (2.2%)	0.480
Yes	0 (0.0%)	9 (100.0%)	

Factors associated with the incidence of a recurrent hernia

The incidence of a recurrent umbilical hernia was significantly higher among females (89.5% vs 19.0%, p < 0.001), as well as those with a BMI of ≥ 25 kg/m^2^ (89.5% vs 62.0%, p = 0.022) and a previous umbilical hernia at the first presentation (89.5% vs 1.3%, p < 0.001); however, it was significantly lower among those who had an obstructed hernia (5.3% vs 30.4%, p = 0.037). A recurrent hernia at the inguinal region was significantly higher among males (89.7% vs 16.7%, p<0.001), those with an obstructed hernia at the first surgery (32.4% vs 10.0%, p = 0.019), and those who underwent a mesh surgery at the first surgery (75.0% vs 53.3%, p = 0.030). Nevertheless, overweight and obese patients had lower rates of inguinal hernias (55.9% vs 93.3%, p < 0.001). Additionally, having a recurrent incisional hernia was significantly associated with the female gender (72.7% vs 27.6%, p = 0.005), having a BMI of ≥ 25 kg/m^2^ (100% vs 63.2%, p = 0.014) and undergoing a non-mesh herniorrhaphy at the first intervention (45.5% vs 28.7%, p = 0.045, Table 3).

**Table 3:** Demographic and hernia-related characteristics at the time of the first intervention

Parameter	Category	Overall, N = 98	BMI < 25, N = 29	BMI 25 or more, N = 69	p-value
Gender	Male	66 (67.3%)	22 (75.9%)	44 (63.8%)	0.244
	Female	32 (32.7%)	7 (24.1%)	25 (36.2%)	
Age (years)	< 16 y	11 (11.2%)	11 (37.9%)	0 (0.0%)	<0.001
	16 to 50 y	37 (37.8%)	6 (20.7%)	31 (44.9%)	
	> 50 y	50 (51.0%)	12 (41.4%)	38 (55.1%)	
Type of hernia	Umbilical hernia	18 (18.4%)	1 (3.4%)	17 (24.6%)	0.013
	Inguinal hernia	71 (72.4%)	28 (96.6%)	43 (62.3%)	<0.001
	Incisional hernia	9 (9.2%)	0 (0.0%)	9 (13.0%)	0.054
Direct or indirect hernia	Direct hernia	43 (43.9%)	15 (51.7%)	28 (40.6%)	0.310
	Indirect hernia	42 (42.9%)	20 (69.0%)	22 (31.9%)	<0.001
Obstructed	Yes	25 (25.5%)	9 (31.0%)	16 (23.2%)	0.416
Type of surgical repair	Mesh	67 (68.4%)	17 (58.6%)	50 (72.5%)	
	Non-mesh herniorrhaphy	30 (30.6%)	12 (41.4%)	18 (26.1%)	
	Not specified	1 (1.0%)	0 (0.0%)	1 (1.4%)	
Time between surgeries	Months	23.2 (13.6, 59.3)	19.8 (11.2, 24.3)	35.0 (15.8, 72.2)	0.006

## Discussion

Baseline differences based on BMI groups

Our analysis found a weak relationship between a high BMI and the recurrence of inguinal hernia, especially indirect hernia. This finding is consistent with those of Burcharth J et al. and Zendejas B et al., who found that BMI did not affect the risk of inguinal hernia recurrence [14,15]. A possible explanation for this might be that excessive fat accumulation within the intra-abdominal space can confer a barrier effect by serving as a physical obstruction that impedes the protrusion of abdominal contents, thus mimicking the properties of a plug [15].

Changes in the type of hernias over time

Also, our research found that overweight and obese patients have a longer time between the first and second interventions for hernia repair than other patients. This could be explained by the fact that overweight and obese patients are advised medically or surgically to reduce their weight for the second optimum intervention.

Current evidence suggests that patients with obesity and hernias may experience better outcomes if they undergo bariatric surgery before or concomitant to ventral hernia repair. Several studies have reported higher rates of complications and recurrence in patients with obesity who undergo hernia repair alone. A retrospective analysis of 106 successive patients who underwent simultaneous bariatric surgery and ventral hernia repair showed they were at a lower risk of recurrence (Krivan et al., 2013) [16]. Another retrospective study of 156 patients reported that bariatric surgery concomitant with mesh ventral hernia repair resulted in fewer complications and lower recurrence rates due to weight loss (Raj et al., 2019) [17]. Therefore, patients with obesity and hernias should consider bariatric surgery before undergoing hernia repair to reduce the likelihood of adverse outcomes. In contrast, another study found that hernia repair at lower BMIs seems suitable, and delaying therapy to lose weight before surgery is probably not advantageous [18]. This result may lead to a longer time interval between the first and second interventions for hernia repair in overweight and obese patients.

In addition, this study also noted that all patients with inguinal and umbilical hernias had the same diagnosis at the second presentation. However, this might be related to poor surgical techniques, including surgeon inexperience, ineffective dissection, insufficient prosthesis size, inadequate prosthesis overlap of hernia defects, incorrect fixation, prosthesis bending, overlooked hernias, or mesh lifting as a result of hematoma development, all of which can contribute to poor results (Lowham et al.) [18]. Furthermore, it could be explained by the high incidence of these types in the Saudi population, which is supported by a local study that found that umbilical and inguinal hernias are the most common types of hernias in the Saudi population, consecutively (Alenazi et al.) [19]. The lack of significant differences in hernia type over time is also essential. This suggests that the risk factors and underlying mechanisms contributing to hernia development and recurrence are likely to be consistent across different types of hernias.

Factors associated with the incidence of a recurrent hernia

Another finding regarding high BMI is a significant increase in the rate of umbilical hernia. These results are consistent with those reported by Shankar DA et al. They also found that BMI is associated with the development of umbilical hernia [20]. It posits that individuals with BMI above 25 kg/m^2^ may be at greater risk for hernia recurrence, potentially due to the additional strain that excess weight places on the abdominal muscles and the stress on the abdominal wall during surgery.

We found that the recurrence rate was significantly lower in patients with an obstructed hernia. This finding contradicts the previous finding by Lau B, which suggested that a BMI greater than 40 kg/m^2^ increased the risk of recurrent hernia obstruction [21]. It seems possible that these results are due to an obstructed hernia that is more likely to develop in minor abdominal defects, which may prevent future recurrences.

Moreover, recurrent inguinal hernias are more common in male patients. This is consistent with the findings of Guillaumes S et al. [22] and Yu et al. [23]. This could be explained by the fact that the existence of a larger and weaker inguinal canal may increase the incidence of inguinal hernia in men compared to women [24].

Our study observed a higher rate of recurrence in patients who underwent mesh repair. This may be attributed to the increased use of mesh repair over alternative techniques [25]. Furthermore, females and obese patients are at higher risk of developing incisional hernia recurrence; this result agrees with Parker et al. [1]. This might be explained by the poor surgical suturing technique, increased intraabdominal pressure, weakened abdominal wall, and multiple pregnancies.

Incisional hernia is less likely to recur in mesh repair rather than in other procedures, which is consistent with Romain B et al. [26]; hence, the proper placement of mesh minimizes the risk of recurrence and complication.

Therefore, patients who undergo hernia repair are often concerned about postoperative pain and the need to return to work and daily activities. Individuals with a history of hernia recurrence may be more susceptible to these concerns. The goal of postoperative pain management is to reduce or eliminate pain and discomfort while minimizing side effects and costs. According to the literature, the combination of IV paracetamol and IV parecoxib is equivalent to the combination of IV paracetamol and intramuscular pethidine for pain relief after open hernia repair [27].

Limitation

Despite its valuable contributions, this study is not restricted. The retrospective design, which relies on medical records, may have introduced incomplete or inaccurate data. In addition, the inclusion of only patients who have undergone hernia repair may lead to selection biases that may underestimate the actual incidence of hernia recurrence in a wider population. Another limitation is the lack of consideration of possible confounding factors, such as smoking and comorbidities, which may affect the risk of hernia recurrence. Finally, the single-institutional setting of the study may limit the generalization of the results to other populations or settings.

## Conclusions

Our study observed that BMI (> 25 kg/m) significantly increases the rate of recurrence of umbilical and incisional hernia while a high BMI is associated with a lower rate of inguinal hernia recurrence. Therefore, we advise weight loss or bariatric surgery for patients with umbilical and incisional hernias only; however, more studies are required to consolidate our findings.
